# Environmental and host plant effects on taxonomic and phylogenetic diversity of root fungal endophytes

**DOI:** 10.1093/femsle/fnaf030

**Published:** 2025-03-03

**Authors:** Emily C Farrer, Nelle K Kulick, Christina Birnbaum, Susannah Halbrook, Caitlin R Bumby, Claire Willis

**Affiliations:** Department of Ecology and Evolutionary Biology, Tulane University, New Orleans, LA 70118, United States; Department of Ecology and Evolutionary Biology, Tulane University, New Orleans, LA 70118, United States; Department of Ecology and Evolutionary Biology, Tulane University, New Orleans, LA 70118, United States; School of Agriculture and Environmental Science, The University of Southern Queensland, Toowoomba, QLD 4350, Australia; Centre for Crop Health, The University of Southern Queensland, Toowoomba, QLD 4350, Australia; Centre for Sustainable Agricultural Systems, The University of Southern Queensland, Toowoomba, QLD 4350, Australia; Department of Ecology and Evolutionary Biology, Tulane University, New Orleans, LA 70118, United States; Department of Ecology and Evolutionary Biology, Tulane University, New Orleans, LA 70118, United States; Department of Ecology and Evolutionary Biology, Tulane University, New Orleans, LA 70118, United States

**Keywords:** coastal marsh, microbiome, *Phragmites australis*, plant-microbe interactions, salinity gradient, *Spartina*

## Abstract

Nearly all plants are colonized by fungal endophytes, and a growing body of work shows that both environment and host species shape plant-associated fungal communities. However, few studies place their work in a phylogenetic context to understand endophyte community assembly through an evolutionary lens. Here, we investigated environmental and host effects on root endophyte assemblages in coastal Louisiana marshes. We isolated and sequenced culturable fungal endophytes from roots of three to four dominant plant species from each of three sites of varying salinity. We assessed taxonomic diversity and composition as well as phylogenetic diversity (mean phylogenetic distance, MPD) and phylogenetic composition (based on MPD). When we analyzed plant hosts present across the entire gradient, we found that the effect of the environment on phylogenetic diversity (as measured by MPD) was host dependent and suggested phylogenetic clustering in some circumstances. We found that both environment and host plant affected taxonomic composition of fungal endophytes, but only host plant affected phylogenetic composition, suggesting different host plants selected for fungal taxa drawn from distinct phylogenetic clades, whereas environmental assemblages were drawn from similar clades. Our study demonstrates that including phylogenetic, as well as taxonomic, community metrics can provide a deeper understanding of community assembly in endophytes.

## Teaser

Environmental conditions and host plant species affect the assembly of fungal endophytes in marsh plant roots.

## Introduction

Plants are colonized by microbial communities that serve as key determinants of plant growth and health (Porras-Alfaro and Bayman 2011, Morelli et al. 2020). Residing in the root tissues, fungal endophytes can function as mutualists promoting nutrient uptake (Vergara et al. 2018, Yakti et al. 2018), disease prevention (Dini-Andreote 2020), and tolerance to abiotic stressors (Jogawat et al. 2016, Yamaji et al. 2016, Gonzalez Mateu et al. 2020). There is increasing interest in restoration and agriculture to use fungal endophytes to enhance plant resilience and crop production, especially in this era of rapid environmental change (Chitnis et al. 2020, Farrer et al. 2022). To better leverage microbial assemblages and their effects on plant health in applied contexts, it is important to understand what drives plant endophyte composition.

One major determinant of endophyte diversity and composition is site-level environmental characteristics. Numerous studies have found that *soil* fungal communities are affected by abiotic site factors, such as salinity (Mohamed and Martiny 2011, Farrer et al. 2021), soil moisture (Zhang et al. 2013), soil nutrient levels (Zhou et al. 2016), and successional stage (Farrer et al. 2019). Because root endophyte communities are primarily recruited from the surrounding soil (Lundberg et al. 2012, Frank et al. 2017), they should be strongly influenced by the composition of the soil microbial species pool. Indeed, studies of *root* fungal communities show that root endophyte composition is affected by factors such as soil salinity (Maciá-Vicente et al. 2012, Hammami et al. 2016, Gonzalez Mateu et al. 2020), site (geographic location) (Glynou et al. 2018), nitrogen (Dean et al. 2014), elevation (Wei et al. 2021), and latitudinal gradients in temperature and precipitation (Glynou et al. 2016).

Host plant identity is another important driver of fungal endophyte communities since host plant traits—root metabolites, exudate chemistry, immune response, productivity, physiology, and root morphology—determine whether endophytes can successfully colonize the plant tissue (Leach et al. 2017, Fitzpatrick et al. 2018, Bergelson et al. 2019, Galindo-Castañeda et al. 2019, Lu et al. 2021). Host species is very important in structuring root fungal endophyte communities within alpine (Dean et al. 2014, Wei et al. 2021, Brigham et al. 2023) and boreal (Kernaghan and Patriquin 2011) ecosystems. Other studies show that the effect of abiotic environment depends on host, with some host species exhibiting variable endophyte assemblages across environments and other host species retaining more consistent assemblages across environments (Maciá-Vicente et al. 2012, Dean et al. 2014). Different host plant genotypes (i.e. native vs. invasive genotypes of *Phragmites*) can also harbor distinct root fungal endophyte communities (Gonzalez Mateu et al. 2020). Consistent with this, in bacterial communities, endosphere community similarity is correlated to the phylogenetic relatedness of the host plants (Fitzpatrick et al. 2018).

Despite these advances toward understanding the structure of root microbial communities, few studies have been placed in a phylogenetic context to understand endophyte community assembly through an evolutionary lens. Understanding phylogenetic diversity, i.e. if a community is composed of highly related or unrelated taxa, is important for both our understanding of biodiversity and for ecosystem management. Recent studies have found that the phylogenetic diversity of root arbuscular mycorrhizal fungi (AMF) increases with plantation age of coffee farms (Aguila et al. 2022), and phylogenetic diversity of leaf-associated fungi increases with successional age in glacial forelands (Matsuoka et al. 2019). If fungal traits are phylogenetically conserved (which may or may not be the case, Kia et al. 2017), phylogenetic diversity can inform mechanisms of community assembly. For example, if communities are more closely related than expected by chance (phylogenetically clustered), habitat filtering may be important in structuring community assembly; whereas if communities are more distantly related than expected by chance (phylogenetically overdispersed), niche partitioning may be important (Webb et al. 2002, Cavender-Bares et al. 2009). Strong phylogenetic clustering has been found in root AMF communities, suggesting the importance of abiotic habitat filtering and host selectivity in these communities (Davison et al. 2016). Another study found that elevated phosphorus increased phylogenetic clustering of root AMF communities, suggesting an increase in host selectivity under these high resource conditions (Frew et al. 2023). Phylogenetic patterns in microbial communities also extend to community composition; for example, one study showed that precipitation affected the taxonomic composition of soil AMF communities but not phylogenetic composition (Chen et al. 2017), suggesting that the differences in composition due to precipitation occurred at the tips of the phylogenetic trees.

Here, we tested how environment and host plant shape fungal root endophyte communities in wetlands. Fungal endophytes in wetland systems are understudied (Lumibao et al. 2024), however, work that has been done suggests both salinity and host species can affect wetland plant endophyte communities (Maciá-Vicente et al. 2012, Gonzalez Mateu et al. 2020). We studied fungal endophytes isolated from roots of 3–4 dominant plants from three coastal marshes in Louisiana ranging from fresh to saline habitats. We hypothesize that both environment and host plant will affect the structure of fungal endophyte communities and that patterns based on phylogenetic relationships (i.e. phylogenetic diversity, phylogenetic composition) will differ from patterns based on taxonomy (i.e. richness, taxonomic composition).

## Materials and methods

### Study sites

**Figure 1.**
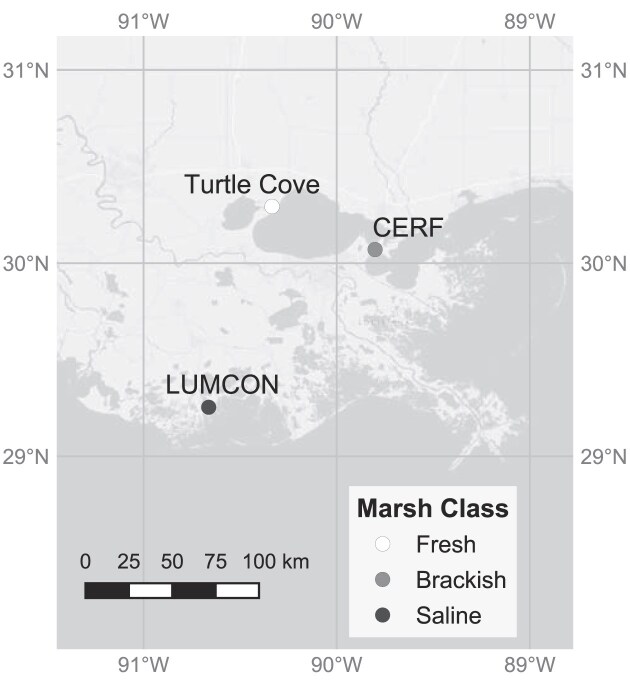
Map of study sites in SE Louisiana, USA. “Turtle Cove” is the Turtle Cove Environmental Research Station, “CERF” is the Coastal Education Research Facility, and “LUMCON” is the Louisiana Universities Marine Consortium.

Samples were collected in July and August of 2017 and 2018 from three coastal marshes arranged along a salinity gradient in southeastern Louisiana (Turtle Cove Environmental Research Station, Coastal Education Research Facility, Louisiana Universities Marine Consortium) (Fig. 1). Marshes were classified as fresh, brackish, or saline based on vegetation and mean annual soil salinities from the three nearest Coastwide Reference Monitoring System (CRMS) and Coastal Wetlands Planning, Protect, and Restoration Act sites to each study location (10 cm depth, 2010–2018).

The freshwater marsh site was located at the Turtle Cove Environmental Research Station (Turtle Cove) in the wetlands of Pass Manchac, Louisiana, a natural pass that connects Lake Pontchartrain to the east with Lake Maurepas to the west (30.293105°N, 90.3353649°W). This site was dominated by *Sagittaria lancifolia* and had a mean annual soil salinity of 1.29 ppt ± 0.47 ppt std. dev. based on CRMS stations 0002-H01, 3650-H01, and 4107-H01 [Coastal Protection and Restoration Authority (CPRA) of Louisiana 2020]. The intermediate/brackish marsh (hereafter “brackish”) was located at the Coastal Education Research Facility (CERF) on the Chef Menteur Pass in East New Orleans, Louisiana, connecting Lake Borgne and the Mississippi Sound to the east with Lake Pontchartrain to the west (30.070006°N, 89.801687°W). This site was dominated by *Spartina alterniflora* and *Spartina patens* with a mean annual salinity of 3.81 ppt ± 1.59 ppt std. dev. based on CRMS stations 0030-H01, 0033-H01, and 0034-H01 [Coastal Protection and Restoration Authority (CPRA) of Louisiana 2020]. The saline marsh site was located at the Louisiana Universities Marine Consortium (LUMCON) in the estuarine wetlands of Cocodrie, Louisiana, adjacent to the Gulf of Mexico, between the Atchafalaya River and Mississippi River deltas (29.253158°N, 90.663280°W). This site was dominated by *S. alterniflora* with a mean annual salinity of 11.39 ppt ± 4.02 ppt std. dev. based on CRMS stations 0434-H01, TE45-H01, and TE45-H02 [Coastal Protection and Restoration Authority (CPRA) of Louisiana 2020]. All sites had well-established monoculture stands of *Phragmites australis* (common reed), a common invader of marshes in coastal Louisiana and along the Gulf Coast.

### Field sampling

Five replicates of 3 to 4 dominant plant species were collected at each site in June 2017 (*n* = 50 plant individuals), and additional samples were collected in July 2018 (*n* = 35 plant individuals). Individual plants of each species were collected at least 2 m apart across the site to avoid collecting clones. Whole plants were dug up, gently washed in water, and then roots were sampled to ensure they came from the correct host plant. *Phragmites australis* (Cav.) Trin. ex Steud. and *S. patens* (Aiton) Muhl. were collected from all sites. The *Phragmites* at the fresh and brackish sites were haplotype I (specifically variant I2, Farrer et al. 2021, and Farrer unpublished data), and at the saline site was haplotype M1 (Farrer et al. 2021). The other species that were collected do not have as wide of a salinity tolerance, so they were not present at all sites. *Sagittaria lancifolia* L. was collected from the freshwater site, *S. alterniflora* Loisel was collected from the brackish and saline site, and *Juncus roemerianus* Scheele was collected from the saline site. Roots were washed in the field to remove excess soil and placed on ice for transport to refrigeration at Tulane University.

### Root endophyte culturing

Root processing and plating were completed within 5 days of collection. Samples were washed under tap water for five minutes at high pressure to remove detritus and soil. Ten 1-cm root samples were selected at random from each plant to maximize culturable endophyte diversity (total *N* plated = 850 root samples). In a sterile laminar flow hood, samples were surface sterilized using 95% ethanol (1 min), 4% bleach (3 min), 95% ethanol (1 min), and sterile water (2 min) (Schulz et al. 1993). Root samples were cut vertically to expose endophytes and plated on 2% malt extract agar (MEA; 20 g of Malt Extract and 20 g of Agar per 1 liter of deionized water) to select for fungi (Kandalepas et al. 2015). To verify the effectiveness of the sterilization method, four uncut samples from each species per site were selected at random and placed on 2% MEA plates for 1 min; nothing grew on these plates. Plated samples and controls were sealed, and fungal endophytes were allowed to grow for 30 days at room temperature, receiving ∼12 h on/off natural light (Clay et al. 2016). To obtain pure fungal cultures, we isolated endophytes by transferring mycelium to fresh MEA plates, allowing them to grow for 14 days, and repeating the process until only a single morphotype was present on each plate. Morphotypes were distinguished by color, shape, margin, surface, opacity, and elevation. To preserve the isolates for reference and potential future use, we photographed each isolate and created two MEA/mycelium vouchers submerged in sterile distilled water in 2.0 ml microcentrifuge vials and two MEA/mycelium slants in 1.5 ml microcentrifuge tubes. These vouchers are stored in the Farrer laboratory at Tulane University.

### Sanger sequencing, taxonomic classification, and phylogenetic methods

We extracted fungal DNA from all isolates using the DNeasy^®^ PowerPlant^®^ Pro Kit (QIAGEN, Germantown, MD, USA) following the manufacturer’s protocols. The ITS-LSU region of the nuclear ribosomal DNA was amplified using TopTaq DNA Polymerase (QIAGEN, USA) in a 20 µl reaction with 2 µl template and primers ITS1F (5′—CTTGGTCATTTAGAGGAAGTAA) and LR3 (5′—GGTCCGTGTTTCAAGAC) (Vilgalys and Hester 1990, Gardes and Bruns 1993). See Supplementary Information for PCR conditions. PCR products were submitted to Genewiz for purification and Sanger sequencing. Forward and reverse sequences were aligned using Mesquite v3.6 (Maddison et al. 2016) and trimmed and edited using Sequencher v5.0 (Gene Codes Corporation, Ann Arbor, MI). These aligned and edited fungal sequences were deposited in NCBI GenBank, organized by host plant species, under accession numbers MN644512-MN644532 (*S. lancifolia*), MN644591-MN644619 (*J. roemerianus*), MN644534-MN644589 (*S. patens*), MN644620-MN644684 (*S. alterniflora*), and MN644685-MN644801 (*P. australis*).

We used the T-BAS: Tree-Based Alignment Selector toolkit v2.3 (Carbone et al. 2019) for phylogenetic-based placement to place sequence data for ITS-partial LSU (ITS1F and LR3 primers) on a fungal reference tree created using six loci (Carbone et al. 2017). T-BAS leverages their reference tree and generates multiple sequence alignments (MSA) that contain the reference and unknown sequences. Their approach allows the reference MSA to include sequences that can be correctly aligned over a portion of their lengths but not alignable in other regions (Carbone et al. 2017). It was developed to work with and has been successfully used with the region amplified by the ITS1F and LR3 primers (Carbone et al. 2017, DeMers and May 2021, Tellez et al. 2022). We used the program’s RAxML de novo multi locus analysis with 100 bootstrap replicates and GTRGAMMA as the rate heterogeneity model. Additionally, we used T-BAS to designate operational taxonomic units (OTUs) on the basis of 97% sequence similarity, and we assigned taxonomy using the UNITE database (Abarenkov et al. 2024). We used FUNGuild (Nguyen et al. 2016) to classify fungal OTUs by putative ecological guild; because the majority of our taxa could not be assigned to a single guild, we could not do further statistical analysis on this data.

### Statistical analysis

Fungal root endophyte diversity was evaluated as OTU richness (number of unique OTUs per individual) and mean phylogenetic diversity (MPD). We used the R (R Core Team 2022) package picante to calculate MPD using the standardized effect size weighted by abundance with the function ses.mpd() (Kembel et al. 2010). This metric provides a measure of phylogenetic diversity by comparing the mean phylogenetic distance between all pairs of individuals in an observed community to that obtained for null communities generated by randomizing species across the tips of the phylogeny and normalizing by the standard deviation of phylogenetic distances in the null communities (Webb 2000, Kembel et al. 2010). MPD essentially gives a metric of phylogenetic diversity controlling for the number of individuals/species in a sample and tree topology by comparing it to null expectations. A mean MPD that does not differ from zero indicates no pattern of relatedness (i.e. randomness) among members within a community. A mean MPD that is greater than zero reflects phylogenetic overdispersion, i.e. co-occurring taxa are more distantly related than expected by chance. A mean MPD that is significantly less than zero reflects phylogenetic clustering, where co-occurring taxa in a community are more closely related than expected at random.

We used two different general linear models to test for effects of explanatory variables on richness and MPD. First, using the full data set, we tested for the effect of host plant and environment (as a factor/categorical variable) on richness and MPD (we could not test for the interaction because not all species were present at all sites). Second, using only the species that were present across the three sites (*P. australis* and *S. patens*), we tested the effects of host plant, environment, and their interaction on richness and MPD. Models were fit using the function lme() in R package nlme (Pinheiro et al. 2023), and a type III ANOVA was used to test for significance of independent variables. Year was used as a random effect to account for any differences in the two collection years.

We also tested whether mean MPDs for each species at each site were different from zero (indicating overdispersion or phylogenetic clustering) using t-tests within the package emmeans (Lenth 2023) and correcting for multiple comparisons using fdr.

We tested the effect of host plant and environment on root endophyte community composition using a taxonomic metric (Bray–Curtis dissimilarity) and a phylogenetic metric (MPD) of composition. Again, we tested two models: (1) using the full data set, we tested the effect of host plant and environment on composition, and (2) using the reduced data set (*P. australis* and *S. patens*), we tested host plant, environment, and their interaction on composition. We used distance-based redundancy analysis (dbRDA) ordination in the R package vegan (Oksanen et al. 2022) and a PERMANOVA permutation test (999 permutations) to test significance of the explanatory variables. Year was used as a conditioning variable in all analyses.

All figures were created using ggplot2 (Wickham 2016).

## Results

### Community description

We cultured a total of 329 fungal endophyte isolates, 151 in 2017 and 178 in 2018. Of these, we obtained 273 high-quality sequences, 128 from 2017 and 145 from 2018. These sequences represent 56 OTUs to which we could putatively assign 4 phyla (majority Ascomycota), 18 orders, 33 genera, and 30 species (see Supplementary Table 1 for the number of isolates and OTUs per plant species at each site). Classification of the sequence data reported a mix of putative pathogenic/parasitic (*Curvularia, Exserohilum, Fusarium, Ilyonectria, Magnaporthaceae, Rhizopus*) and putative commensal/mutualistic (*Acephala, Mortierella, Xylaria, Buergenerula, Paraconiothyrium, Sarocladium*) symbionts.

### Diversity

**Figure 2.**
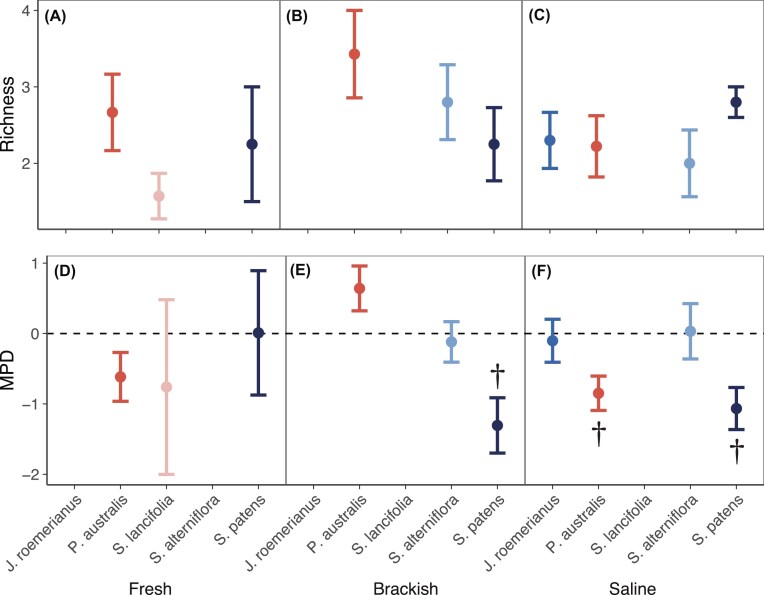
Endophyte richness (A–C) and phylogenetic diversity (MPD, D–F) in different host plants and environments. Error bars represent means ± 1 SE. For phylogenetic diversity, negative MPD values indicate phylogenetic clustering, and positive MPD values indicate overdispersion. Symbols denote mean MPD significantly different from zero (corrected for multiple comparisons): † *P* < .1.

Neither host plant nor environment significantly affected the richness of root fungal communities (Fig. 2A–C). Similarly, when analysis was done on a reduced dataset including only those host plants that were present across all sites (*P. australis, S. patens*), there was no effect of host plant, environment, or their interaction.

Phylogenetic diversity (as measured by MPD) was likewise not affected by host plant or environment in the full dataset; however, when only *P. australis* and *S. patens* were analyzed, we found that the effect of environment on phylogenetic diversity depended on host (significant host × environment interaction, *F*_2,22_ = 5.16, *P* = .015). Specifically, for *P. australis* phylogenetic diversity was <0 only at the saline site, but for *S. patens* phylogenetic diversity was <0 at the brackish and saline sites (Fig. 2D–F). An MPD <0 is indicative of phylogenetic clustering.

### Composition

**Figure 3.**
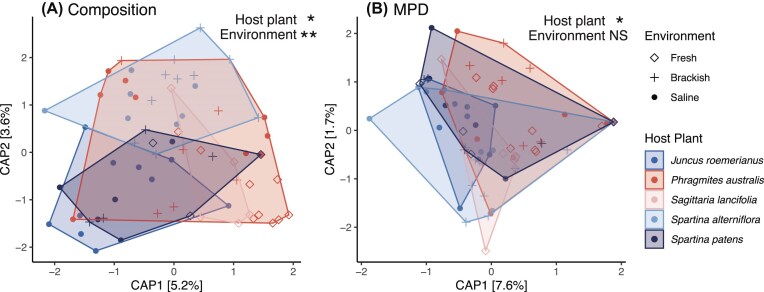
Distance-based RDAs show the effect of environment (symbol) and host plant (color) on taxonomic composition (measured by Bray–Curtis dissimilarity) (A) and phylogenetic composition (measured by abundance-weighted mean phylogenetic distance) (B) of root endophyte communities. Symbols denote significance of permutation (PERMANOVA) tests: *P < .05, **P < .01, NS = not significant; see Table 1 for full permutation test results.

Both host plant and environment significantly affected the taxonomic composition (as measured by Bray–Curtis dissimilarity) of endophyte communities for the full dataset as well as for the reduced dataset including only *P. australis* and *S. patens* (Fig. 3A, Table 1). Interestingly, only host plant (not environment) affected phylogenetic composition (as measured by MPD) for both the full dataset and the reduced dataset, suggesting that different host plants selected for fungal taxa that were drawn from distinct phylogenetic clades (Fig. 3B, Table 1).

**Table 1.** Results from dbRDA permutation tests (PERMANOVA), testing the effect of host plant, environment, and (for the *P. australis* and *S. patens* models) their interaction on cultured root endophyte communities of marsh plants.

Dependent variable	Model	Explanatory variable	Variance explained	Pseudo-*F* (df)	*P*
Taxonomic composition (Bray–Curtis)	Full model	Host plant	7.1%	1.32 (4, 61)	.018*
		Environment	4.6%	1.71 (2, 61)	.003**
	*P. australis* and *S. patens*	Host plant	6.6%	2.86 (1, 33)	<.001***
		Environment	9.8%	2.13 (2, 33)	<.001***
		Host plant × env	5.7%	1.26 (2, 31)	.114
Phylogenetic composition (MPD)	Full model	Host plant	8.3%	1.59 (4, 64)	.045*
		Environment	2.2%	0.86 (2, 64)	.566
	*P. australis* and *S. patens*	Host plant	14.2%	6.51 (1, 33)	<.001***
		Environment	6.7%	1.52 (2, 33)	.134
		Host plant × env	3.8%	0.85 (2, 31)	.534

Year was used as a conditioning variable in all ordinations. See Fig. 3 for ordination plots.

## Discussion

Many different drivers can contribute to patterns of taxonomic and phylogenetic diversity of plant endophytes. Here, we found no effect of environment or host on the taxonomic richness of root endophytes across a marsh salinity gradient. However, we found that the effect of environment on phylogenetic diversity depended on host plant, such that different host plants had different patterns of phylogenetic diversity at different sites. We also found evidence of phylogenetic clustering for some of the plant species across the gradient suggesting that habitat filtering may be structuring fungal endophyte communities. Both environment and host plant strongly affected taxonomic composition of the fungal communities, but only host plant affected phylogenetic composition. Overall, this indicates that both environment and host plant structure fungal root endophyte communities, and some differences exist when assessing patterns with a taxonomic vs. phylogenetic metric, which can give us insights into characteristics and processes occurring in these microbiomes.

We found an average of 2–3 fungal taxa per individual plant sample in our study (8–30 taxa per plant species), which is similar to what is found in other culture-based studies (Kernaghan and Patriquin 2011, Maciá-Vicente et al. 2012, Clay et al. 2016, Kimbrough et al. 2019, Høyer and Hodkinson 2021). The taxa we recovered are common symbionts in wetland plant communities, including the genera *Sarcocladium, Fusarium, Septoriella, Aureobasidium, Mortierella, Sarocladium, Talaromyces*, and *Phaeosphaeria* (Kandalepas et al. 2015, Clay et al. 2016). The most common species were *Trichoderma harzianum* and *Paraconiothyrium estuarinum. Trichoderma harzianum* is widely distributed across many ecosystems including wetlands (Saravanakumar et al. 2016), and is commonly used in agriculture as a biocontrol agent against plant pathogens (Poveda et al. 2019). *Paraconiothyrium estuarinum* has been isolated from estuarine/wetland sediments (Verkley et al. 2004) and forage grasses (Martins Alves et al. 2021) and has been found to be able to degrade polycyclic aromatic hydrocarbons (Verkley et al. 2004), inhibit pathogen growth, and promote plant growth (Martins Alves et al. 2021).

### Taxonomic diversity and composition

We found no effect of host plant or environment on taxonomic richness, but we did find differences in taxonomic composition, a pattern also found in two other endophyte studies across a salinity gradient (Hammami et al. 2016, Gonzalez Mateu et al. 2020). This suggests that salinity, as a stress, does not necessarily limit the diversity of microbes in plant roots, but just changes their composition. Likewise, host plant species may not differ in fungal endophyte diversity but they do differ in taxonomic composition, as has been found in boreal trees (Kernaghan and Patriquin 2011). The lack of effects on richness may not be surprising in a culture-dependent study since the richness of cultured endophytes is generally low. However, other studies (Dean et al. 2014, Wei et al. 2021), including a culture-dependent study (Lyons et al. 2021), have found that some environments and plant species can host a higher diversity of endophytes than others. The strong host and environment effects on endophyte taxonomic composition found here are consistent with many studies that find environment (Maciá-Vicente et al. 2012, Hammami et al. 2016, Gonzalez Mateu et al. 2020) and host plant species (Kernaghan and Patriquin 2011, Dean et al. 2014, Lyons et al. 2021, Wei et al. 2021) structure fungal endophyte composition. Environmental effects on endophyte composition are perhaps not surprising; even though living within the host plant may shield the endophyte from stressful abiotic conditions, most endophytes are horizontally transmitted and many have free-living lifestyles (Bard et al. 2024) that would require tolerance of the abiotic environmental conditions in the habitat. Host species effects on endophyte composition are also expected, especially as our host species are distantly related (in three different plant families) (Glynou et al. 2016), and thus likely differ in their chemistry, morphology, and immunity genes.

### Phylogenetic diversity and composition

The phylogenetic perspective explored here brings a deeper understanding to fungal endophyte community structure and assembly. While other studies have shown that host species (Matsuoka et al. 2021) and environment (Matsuoka et al. 2019) can affect phylogenetic diversity of litter-associated fungal communities and host functional group (Davison et al. 2020) and environment (Aguila et al. 2022) can affect phylogenetic diversity of root AMF communities, few studies test multiple hosts across multiple environments. Our results showed that the effect of environment on phylogenetic diversity depended on species, with *P. australis* having the highest phylogenetic diversity in the brackish marsh and *S. patens* having the highest phylogenetic diversity in the fresh marsh. Because phylogenetic diversity can affect multifunctionality (Delgado-Baquerizo et al. 2016, Le Bagousse-Pinguet et al. 2019) and has been used as a proxy for functional diversity in microbes (Davison et al. 2016), this might suggest that different plants require or experience different levels of multifunctionality from their endophytes in different environments.

The phylogenetic clustering (MPD < 0) observed in three instances (*S. patens* brackish, *S. patens* saline, *P. australis* saline) is consistent with other studies that generally find phylogenetic clustering (rather than overdispersion) of root endophytes (Maciá-Vicente and Popa 2022), AMF communities (Davison et al. 2016), root sebacinoid (Basidiomycota: Agaricomycetes) fungi (Garnica et al. 2013), and leaf endophytes (Del Olmo-Ruiz and Arnold 2017, Lumibao et al. 2019). There is evidence that at least some traits may be phylogenetically conserved in fungal endophytes (Kia et al. 2017), AMF (Powell et al. 2009), and microbes in general (Martiny et al. 2015). If we assume some phylogenetic conservatism of fungal traits, then phylogenetic clustering suggests that host and environmental filtering are structuring endophyte community assembly by selecting for taxa with similar, adaptive traits. Our finding that phylogenetic clustering in root endophytes can change across salinity gradients for some species is consistent with Frew et al. (2023), who found that phylogenetic clustering in *Sorghum* AMF communities increases across a phosphorus gradient. Plant species may differ in selectivity (greater phylogenetic clustering) of endophytes depending on the stresses they experience across environmental gradients (Frew et al. 2023). Interestingly, we found more phylogenetic clustering at the saline end of the gradient, which might suggest that both *P. australis* (which is abundant across the gradient) and *S. patens* (which is rare at high and low salinity) may benefit from selectivity under stress.

We found that host plant affected phylogenetic composition, but environment did not. This suggests that different host plants draw their communities from distinct phylogenetic clades, but that environmental assemblages (which are taxonomically different, see above) are drawn from similar clades. In other words, environmental assemblages differed only at the tips of the phylogenetic tree. This is consistent with another recent study that found host species affects phylogenetic composition of root fungal communities in bromeliads (Leroy et al. 2021). However, our results contrast with those from another study that found different tropical forest sites (which differed in precipitation, elevation, and fragmentation) differed in phylogenetic composition of leaf endophytes (Del Olmo-Ruiz and Arnold 2017). It might be that salinity is relatively easy for fungi to adapt to compared to other environmental stressors, and laboratory evolution studies have shown that some fungal taxa can adapt to tolerance of higher salinities over time (Jones et al. 2022).

### Limitations

While this is an important first step in understanding root fungal assembly across different hosts and environments, there are some limitations to our study. First, this is a culture-dependent study, and it is well known that only a small percentage (estimated at 10%) of fungal diversity is culturable (Wu et al. 2019). Furthermore, our sample sizes were rather small, and we only sampled a subset of the root system; thus, we likely did not capture the full biodiversity of fungi in our host plants (Supplementary Table 1). Future work utilizing culture-independent data and the ghost tree approach is a promising direction for studying phylogenetic patterns in fungi (Fouquier et al. 2016). Secondly, we only sampled one site per salinity regime, and as endophyte biodiversity patterns and drivers can differ across sites (Alzarhani et al. 2019), future studies should aim to sample more replicated locations.

### Implications and conclusions

Elucidating the drivers of endophyte assembly is important for our understanding of the microbial biodiversity that impacts plant health, and a phylogenetic perspective can deepen our understanding of microbial systems. Here, we show that both environmental characteristics and host plant identity affect composition of root fungal microbiomes, but that communities in different salinity environments only differed at tips of the phylogenetic tree while host microbiomes differed at a more basal level. Phylogenetic analysis also indicated phylogenetic clustering, which suggests that host and habitat filtering (rather than competition) are important in structuring root fungal communities. Understanding that environment and host species affect root microbiomes is important to applied work in restoration and agriculture that may seek to inoculate plants with novel endophytes to promote plant growth; our work suggests that sourcing endophytes from similar hosts and environments may yield the highest inoculation success. Our work also predicts that notable shifts in microbiomes will occur in the near future with increasing saltwater intrusion and salinization in coastal areas worldwide. Overall, more study of fungal microbiomes is critical to understand and ensure plant resilience, particularly in ecosystems such as coastal wetlands that are at the frontlines of global change impacts.

## Supplementary Material

fnaf030_Supplemental_File

## Data Availability

The ITS1-LR3 sequence data were deposited in the NCBI GenBank under accession numbers MN644512-MN644532 (*S. lancifolia*), MN644591-MN644619 (*J. roemerianus*), MN644534-MN644589 (*S. patens*), MN644620-MN644684 (*S. alterniflora*), and MN644685-MN644801 (*P. australis*). Processed data and metadata files (Farrer et al. 2025) are available through the Environmental Data Initiative (EDI) at https://doi.org/10.6073/pasta/06e760e23c3a288fc669f40ce53871c9. Code is available on GitHub at https://github.com/ecfarrer/LAmarshCulture2.
