# Supplementary material for: Environmental and host plant effects on taxonomic and phylogenetic diversity of root fungal endophytes
Source: FEMS Microbiol Lett. 2025 Mar 3;372:fnaf030. doi: 10.1093/femsle/fnaf030 (PMC11895511; doi:10.1093/femsle/fnaf030)
Supplement: fnaf030_Supplemental_File [file fnaf030_supplemental_file.docx]

**Supplementary Information**

**Environmental and host plant effects on taxonomic and phylogenetic diversity of root fungal endophytes**

**PCR conditions.**
Each PCR reaction mixture included 13.875 µL of UltraPure Distilled Water (Invitrogen by Thermo Fisher Scientific, Grand Island, NY, USA), 2.5 µL of 10X TopTaq buffer (contains 15 mM MgCl2) (QIAGEN, Hilden, Germany), 0.5 µL of 10 mM dNTPs, 0.5 µL of 10 mM of each forward and reverse primers, and 0.125 µL of x units TopTaq polymerase. For PCR reactions, we used Eppendorf Mastercycler pro S Thermal Cycler (Eppendorf, Hauppauge, NY, USA) with following conditions: 94°C for 3 min; 34 cycles of 94°C for 30 sec, 54 °C for 30 sec, 72 °C for 1 min; and 72 °C for 10 min. PCR products were confirmed by visualization with SYBR Safe DNA gel stain (Invitrogen by Thermo Fisher Scientific, Grand Island, NY, USA) on 1.5% agarose gels.

**Supplementary Table 1.** The number of fungal isolates and OTUs obtained from our sampling. The # isolates (2^nd^ column) indicates the number of isolates cultured from each plant individual. The # individuals successfully cultured (3^rd^ column) indicates the number of individuals that grew isolates; a few individuals that we collected got contaminated or grew no fungi. The # OTUs (4^th^ column) indicates the number of OTUs obtained from samples based on a 97% similarity threshold. The # individuals successfully cultured and sequenced (5^th^ column) indicates the number of individuals from which we got high quality sequences.

|  | # Isolates | # Individuals successfully cultured | # OTUs | # Individuals successfully cultured and sequenced |
| --- | --- | --- | --- | --- |
| Means per individual sampled (± SE) |  |  |  |  |
| Fresh |  |  |  |  |
| *Phragmites australis* | 5.90 (± 0.85) | 10 | 2.67 (± 0.50) | 9 |
| *Sagittaria lancifolia* | 2.56 (± 0.63) | 9 | 1.57 (± 0.30) | 7 |
| *Spartina patens* | 3.75 (± 1.55) | 4 | 2.25 (± 0.75) | 4 |
| Brackish |  |  |  |  |
| *Phragmites australis* | 4.44 (± 1.03) | 9 | 3.43 (± 0.57) | 7 |
| *Spartina alterniflora* | 4.45 (± 0.72) | 11 | 2.80 (± 0.49) | 10 |
| *Spartina patens* | 5.50 (± 1.19) | 4 | 2.25 (± 0.50) | 4 |
| Saline |  |  |  |  |
| *Juncus roemerianus* | 3.27 (± 0.41) | 11 | 2.30 (± 0.37) | 10 |
| *Phragmites australis* | 4.00 (± 1.05) | 9 | 2.22 (± 0.40) | 9 |
| *Spartina alterniflora* | 2.78 (± 0.86) | 9 | 2.00 (± 0.44) | 7 |
| *Spartina patens* | 4.80 (± 0.97) | 5 | 2.80 (± 0.20) | 5 |
|  |  |  |  |  |
| Totals by site or by host plant |  |  |  |  |
| Fresh | 97 | 23 | 20 | 20 |
| Brackish | 111 | 24 | 33 | 21 |
| Saline | 121 | 34 | 35 | 31 |
| *Juncus roemerianus* | 36 | 11 | 16 | 10 |
| *Phragmites australis* | 135 | 28 | 30 | 25 |
| *Sagittaria lancifolia* | 23 | 9 | 8 | 7 |
| *Spartina alterniflora* | 74 | 20 | 20 | 17 |
| *Spartina patens* | 61 | 13 | 27 | 13 |
